# Multiplexed Affinity Measurements of Extracellular Vesicles Binding Kinetics

**DOI:** 10.3390/s21082634

**Published:** 2021-04-09

**Authors:** Elisa Chiodi, George G. Daaboul, Allison M. Marn, M. Selim Ünlü

**Affiliations:** 1 Department of Electrical Engineering, Boston University, Boston, MA 02215, USA; ammarn@bu.edu (A.M.M.); selim@bu.edu (M.S.Ü.); 2 NanoView Biosciences, Boston, MA 02135, USA; gdaaboul@nanoviewbio.com; 3 Department of Biomedical Engineering, Boston University, Boston, MA 02215, USA

**Keywords:** label-free biosensor, extracellular vesicles (EVs), EVs detection, microarray, interferometric imaging

## Abstract

Extracellular vesicles (EVs) have attracted significant attention as impactful diagnostic biomarkers, since their properties are closely related to specific clinical conditions. However, designing experiments that involve EVs phenotyping is usually highly challenging and time-consuming, due to laborious optimization steps that require very long or even overnight incubation durations. In this work, we demonstrate label-free, real-time detection, and phenotyping of extracellular vesicles binding to a multiplexed surface. With the ability for label-free kinetic binding measurements using the Interferometric Reflectance Imaging Sensor (IRIS) in a microfluidic chamber, we successfully optimize the capture reaction by tuning various assay conditions (incubation time, flow conditions, surface probe density, and specificity). A single (less than 1 h) experiment allows for characterization of binding affinities of the EVs to multiplexed probes. We demonstrate kinetic characterization of 18 different probe conditions, namely three different antibodies, each spotted at six different concentrations, simultaneously. The affinity characterization is then analyzed through a model that considers the complexity of multivalent binding of large structures to a carpet of probes and therefore introduces a combination of fast and slow association and dissociation parameters. Additionally, our results confirm higher affinity of EVs to aCD81 with respect to aCD9 and aCD63. Single-vesicle imaging measurements corroborate our findings, as well as confirming the EVs nature of the captured particles through fluorescence staining of the EVs membrane and cargo.

## 1. Introduction

In the past few years, the interest in Extracellular Vesicles (EVs) as theranostic tools has significantly increased [1]. These biological particles constitute a very heterogeneous population in the human body, in both origin and size. They range from vesicles of endosomic origin (small EVs, or exosomes, 50–150 nm) to microvesicles (50 nm–1 μm) released from the plasma membrane [2]. The heterogeneity of these biological nanoparticles can sometimes pose a challenge in terms of purification and phenotyping.

When they were first discovered, EVs were considered to be a cellular discard. They soon revealed their potential, proving to be a valuable asset in the field of biomarker discovery and therapy design. By carrying pieces of information in the form of RNA fragments and biomarkers, EVs act as a “journal" of each individual’s health conditions and make a crucial contribution to the intracellular communication.

One of the main challenges of research on EVs is the time-consuming nature of experiments including laborious purification processes. Moreover, in order to get accurate results, maximizing binding efficiency of the vesicles to their specific probe is a necessity; for end-point measurements, this implies ensuring the saturation of the capture reaction, which often results in an unnecessarily long incubation time. A typical phenotyping experiment involves a 12 h or overnight incubation of the sensor chips with the EVs sample, thus causing a significant delay in data acquisition and interpretation. In order to exploit the potential utilization of EV biomarkers, faster and high-throughput analysis methods and tools are needed.

To address this need, a variety of real-time detection methods for extracellular vesicles have been studied [3,4]. For example, it has been demonstrated that a good technique to monitor the behavior of EVs in real time is to label them with either a fluorescent probe (Real-Time Fluorescence Microscopy Single Particle Tracking (SPT) [5]) or a gold nanoparticle [6,7,8]. Labeling offers great sensitivity, and tagging with fluorescent/gold-labeled anti-tetraspanins ensures the specificity of the tracked particle. However, it is an indirect method that can be prone to artifacts such as non-specific binding and photo-instability of the fluorescent molecules, or alteration of the binding affinity due to Au labels with larger mass than the EV itself.

Another established method for the real-time detection of extracellular vesicles is Surface Plasmon Resonance Imaging (SPRi) [9,10,11,12]. This technique succeeds in monitoring the label-free binding of EVs to different probes simultaneously. However, as in the case of all evanescent-wave based sensors, SPR cannot distinguish surface binding from local changes in solution refractive index, and it is prone to environmental factors (temperature, vibrations, pH variations). Since EVs are normally purified from plasma or cell culture media, the typical target solutions are highly heterogenous. Therefore, the large variations in refractive index further exacerbate the background noise in SPR measurements.

In this work, we developed a whole new application for kinetic measurements on Interferometric Reflectance Imaging Sensor by tailoring it to the multiplexed phenotyping of EVs. By quantifying the thickness accumulated on the surface, and by removing any unwanted background effects through differential measurements, we were able to precisely evaluate the dynamic accumulation of EVs. Through the use of low-magnification optics, we combined real-time detection and multiplexing, simultaneously measuring the real-time capture of EVs on three distinct antibody surfaces (aCD9, aCD63, aCD81), each immobilized at six different concentrations. This way, we could efficiently optimize the probe density, probe specificity, incubation time, and flow velocity in a single experiment. The acquisition time was maintained below one hour per experiment, therefore yielding a reliable, quantitative, and easy optimization method for EVs characterization.

The kinetic results obtained with the IRIS were corroborated by single-particle measurements, both label-free and labeled, that were performed on the ExoView™ system (Section 3.1).

## 2. Materials and Methods

### 2.1. EVs Culture and Purification

HEK293T cell line was cultured using Dulbecco’s modified Eagle’s medium (DMEM) (Thermo Fisher Scientific, Waltham, MA, USA) supplemented with 10% (*v/v*) of FBS (Gibco, Frederick, MD, USA, and Invitrogen, Carlsbad, CA, USA). Upon confluence, the cells were washed twice with dPBS (Gibco, Frederick, MD, USA and Invitrogen, Carlsbad, CA, USA) and incubated in DMEM supplemented with 10% (*v/v*) of exosome-depleted FBS (Gibco, Frederick, MD, USA and Invitrogen, Carlsbad, CA, USA) for 48 h. Conditioned media were then collected and centrifuged at 2500× *g* for 15 min at room temperature to remove cellular debris. The supernatant was transferred to a new tube and was centrifuged at 2500× *g* for 15 min at room temperature. Finally, the supernatant was aliquoted and stored at −80 ${}^{\circ }$C until usage.

EVs were concentrated through ultracentrifugation to allow binding measurements on the IRIS system. To concentrate the EVs, 60 mL of conditioned media was pelleted by ultracentrifugation at 110,000× *g* for 16 h at 4 ${}^{\circ }$C in S50A Rotor (Thermo Scientific). The pellet was resuspended into 60 mL of 1XPBS and pelleted again at 110,000× *g* for 6 h at 4 ${}^{\circ }$C. The pellet was then resuspended in 0.5 mL of PBS.

Prior to the IRIS experiment, the EVs sample was centrifuged at 60,000 rpm for 10 min to eliminate large aggregates. The supernatant was then diluted (2X) to be used for the experiment, while the pellet was discarded.

### 2.2. The IRIS Platform

The Interferometric Reflectance Imaging Sensor (IRIS) (Figure S1, in the Supplementary Materials) has been extensively described in many publications [13]. Briefly, a silicon chip with a layer of thermally grown silicon oxide (SiO_2_) on top is used as a substrate, where biomolecules are immobilized through an active surface chemistry (described in Section 2.5).

The Si/SiO_2_ substrate is illuminated from the top through a microfluidic chamber, and it acts as a common path interferometer, where light reflecting from top of the sensor surface interferes with the reference reflection at the oxide-Si interface. Accumulation of biological mass on the sensor surface increases the effective thickness from the top surface to the reference Si surface and alters the reflectance. In IRIS measurements, the molecular weight of the analyte can vary from small molecules [14] to macroparticles such as viruses [15]. The reflectance coming from the surface assumes the form:

(1) $$R=|r^{2}|=\frac{{r_{1}}^{2}+{r_{2}}^{2}+2r_{1}r_{2}\cos2\phi }{1+{r_{1}}^{2}{r_{2}}^{2}+2r_{1}r_{2}\cos2\phi }$$

where $\phi =\frac{2\pi d}{\lambda }n_{SiO_{2}}$.

The oxide thickness is engineered to provide maximum constructive interference in response to biomass accumulation, therefore resulting in an increase in signal from the active probe spots. By generating a lookup Table [16], it is possible to convert this signal difference to mass per unit area, therefore allowing for quantitative surface density measurements. The 50-μm thick microfluidic chamber allows for high flow rates and reduction of mass transport effect.

### 2.3. The ExoView™ System

The single particle characterization experiments of the EVs samples were carried out on the ExoView™ system by NanoView, BioSciences.

### 2.4. Simulations, Data Acquisition and Analysis

Reflectance simulations providing the change in reflectance due to the accumulation of EVs on the sensor surface were performed in MATLAB.

The real-time images were acquired through Micro-Manager software. Between subsequent frames, a fixed interval of 60 ms was always maintained, and 100 frames were averaged to obtain one image. Therefore, all data points were 6 s-spaced. Taking into account the results of the reflectance simulations, the videos could be converted to mass per unit area by applying a look-up table to the images, through a custom MATLAB code that uses the bare silicon region as a normalization reference. The images were then analyzed in ImageJ. A donut-shaped region of film around each spot was used as a background, and the differential mass density data thus obtained were plotted and fitted in MATLAB. For the IgG1 control experiment, a bivalent fitting model was applied. On the other hand, to fit the binding curves of exosomes, a more complex model was utilized, where a combination of fast and slow association and dissociation rates were considered [17], as further detailed in Section 4.2.

### 2.5. Surface Chemistry and Chip Spotting

Prior to probe immobilization, chips were coated with MCP-2 polymer (Lucidant). This reactive polymer is based on DMA-NAS-MAPS chemistry and has been extensively described in the literature [18].

To fabricate chips with different antibody density on the surface, the tetraspanin-specific antibody was mixed with Mouse IgG2a. The total concentration of the spotted antibody was maintained at 3 mg/mL; however, the ratio of tetraspanin-specific antibody (CD81, CD63, and CD9) and Mouse IgG2a was varied. The antibody solutions were spotted onto the polymer coated chips using S12 sciFLEXARRAYER from ScienIon. The antibodies were allowed to immobilize on the surface for four hours in a humid chamber. Then, the chips were washed in 1X PBST followed by a rinse in Millipore water and carefully dried under nitrogen stream.

### 2.6. Antibodies and Other Reagents

Antibodies used for making the chips and antibody labeling were anti-CD81 (JS-81), anti-CD63 (H5C6), anti-CD9 (HI9a), Mouse IgG1k, and Mouse IgG2a provided by NanoView Biosciences.

## 3. Results

### 3.1. EVs Characterization by Single Particle Interferometric Imaging

**Figure 1:**
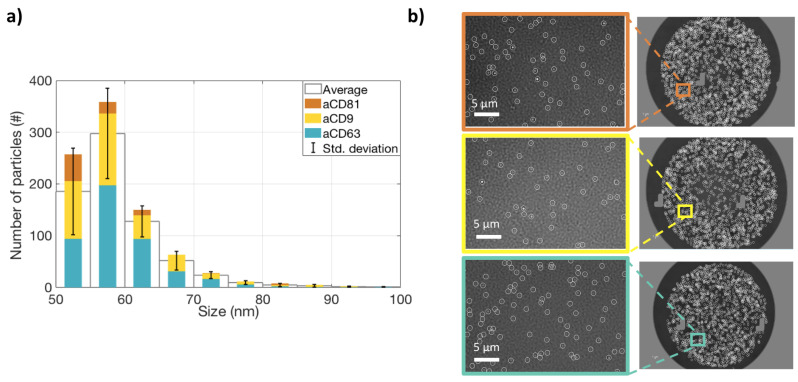
Label-free measurements of extracelluralar vesicles’ (EVs’ )shape and size distribution. (**a**) The size distribution of EVs captured on CD9, CD81, and CD63 spots. The colored bars represent the number of particles counted on each antibody spot. The white bars represent the average of the three antibodies, and the black lines show the standard deviation. (**b**) Label-free images of the particles on the three spots.

**Figure 2:**
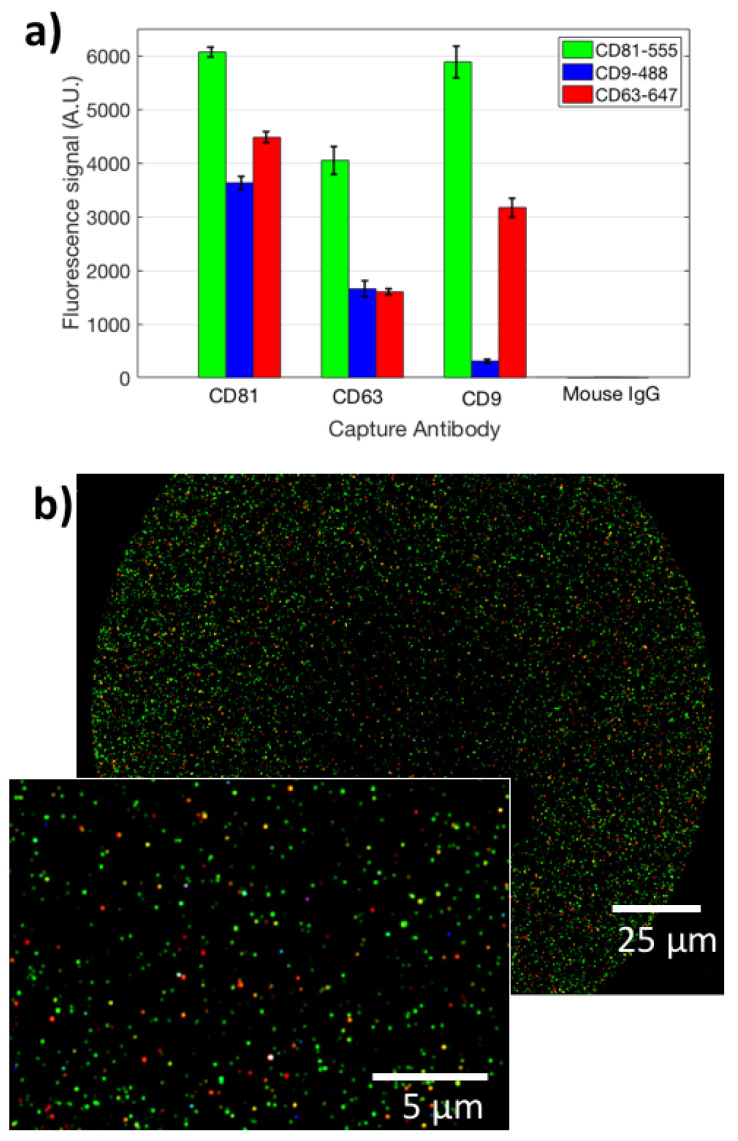
(**a**) The fluorescence signal obtained for the analyzed EVs from staining with three different labeled antibodies (aCD81-555, aCD9-488, aCD63-647), on three different capture spots (aCD9, aCD81, aCD63). (**b**) A co-localized fluorescence image of EVs captured on immobilized aCD9. Green points refer to CD81 staining, blue to CD9, red to CD63. The inset shows a zoomed-in image of the same spot.

In order to satisfy the Minimal Information for Studies on Extracellular Vesicles (MISEV 2018 [19]) requirements, we characterized the studied EVs for size, protein content, and surface properties. All the measurements were carried out on the ExoView system. To characterize the vesicles for their size, an ultra-centrifugated (UC) HEK EVs sample was diluted 1:100 and incubated on an SP-IRIS chip in order to capture EVs on three different antibody spots: CD9, CD63, and CD81. Label-free images of the capture spots were acquired on the ExoView system, and counting and sizing of the particles was performed (Figure 1a). The size distribution is coherent with what expected for small EVs. A label-free image of the vesicles is shown in Figure 1b. Subsequently, the same chip was also stained with fluorescent antibodies for CD9, CD81, and CD63, and co-localization was determined (Figure 2a,b). This allowed us to characterize the EVs in terms of their surface receptors. Co-localization has been determined to be high (>50 %) for all the antibodies.

Moreover, to analyze the EVs internal cargo, we stained the 2000X-diluted UC pellet with Anti-Syntenin-555, Anti-CD63-647, Anti-CD9-488 and Anti-CD81-488. Here, the cargo is only stained by Anti-Syntenin-555, while Anti-CD9-488 and Anti-CD81-48 are again staining the membrane. This allows us to demonstrate co-localization of cargo-specific labels and membrane-specific ones, proving the integrity of the vesicles. Internal cargo staining data are reported in Figure S2a. From this figure, it can be noted that there is a good correlation between capturing of the surface probe and internal cargo information that is specific for EVs. Co-localization is high in this case as well. Composite images that show co-localization are displayed in Figure S2b. These preliminary data confirm the small-EVs nature of the studied nanoparticles.

### 3.2. Antibody Density Verification

**Figure 3:**
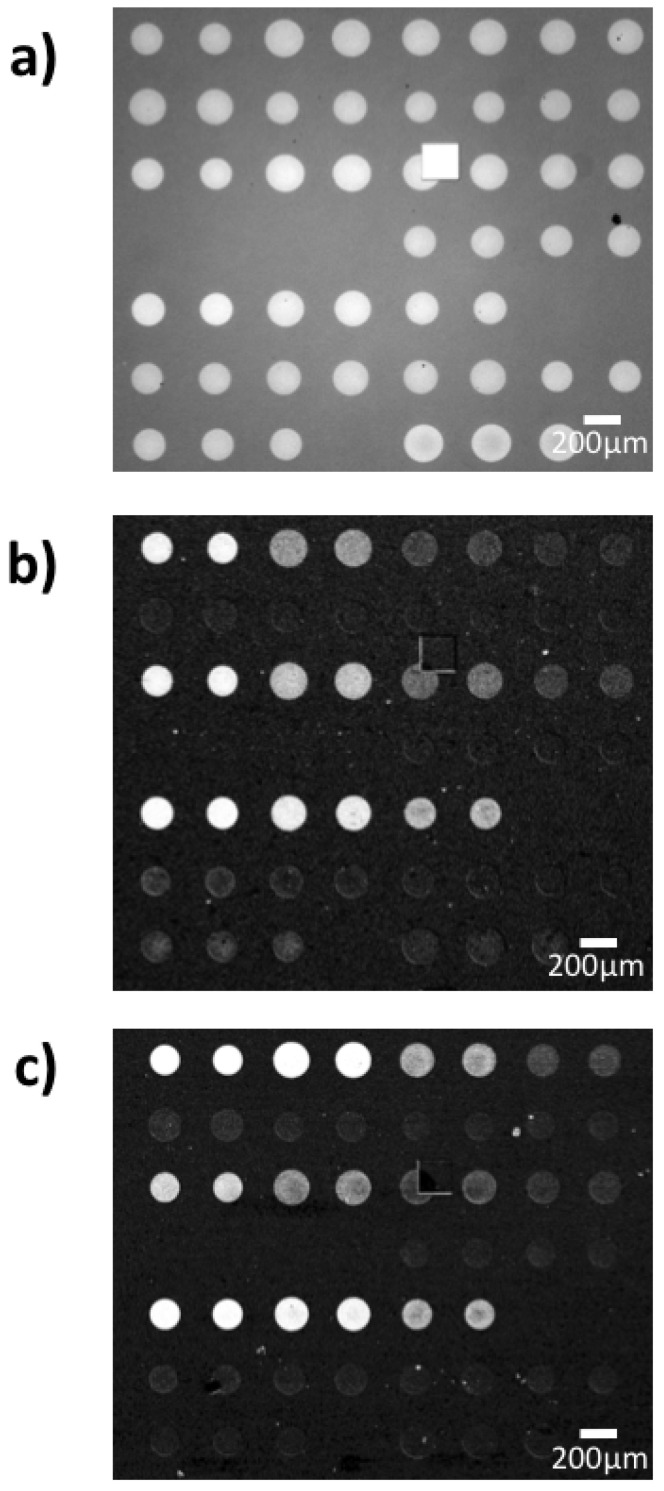
Interferometric Reflectance Imaging Sensor (IRIS) images of the chips utilized for the experiment (**a**) before incubation and differential images after incubation with (**b**) antibodies and (**c**) small EVs. Note that a similar level of signal does not necessarily correspond to the same increase in thickness, since the antibody experiment was carried out with blue LED illumination (452 nm) while the exosome capture was performed with green LED illumination (512 nm), to ensure a proper correlation between signal and thickness.

The probes for the binding kinetics IRIS experiments were spotted in a microarray modality, and an IRIS image of the chip prior to the experiment is reported in Figure 3a. As described in Section 2.5, to maintain a good spot conformation, the density of the active antibodies was tuned by keeping the same total concentration of the spotting solution (3 mg/mL) while varying the percentage of the active molecules. Therefore, even though the surface biomass density was maintained constant for all spots, resulting in similar intensities for all spots in Figure 3a, the percentage of active antibodies within each spot ideally should have changed linearly depending on the IgG1/IgG2 ratio. However, since the yield of the immobilization process is not ideal, the actual amount of active probe within each spot was measured through a control experiment, by flowing a generic IgG1 antibody across the surface of a chip belonging to the same spotting batch as the one used for the EVs detection experiment. A differential image of the IRIS chip after the control experiment can be observed in Figure 3b.

**Figure 4:**
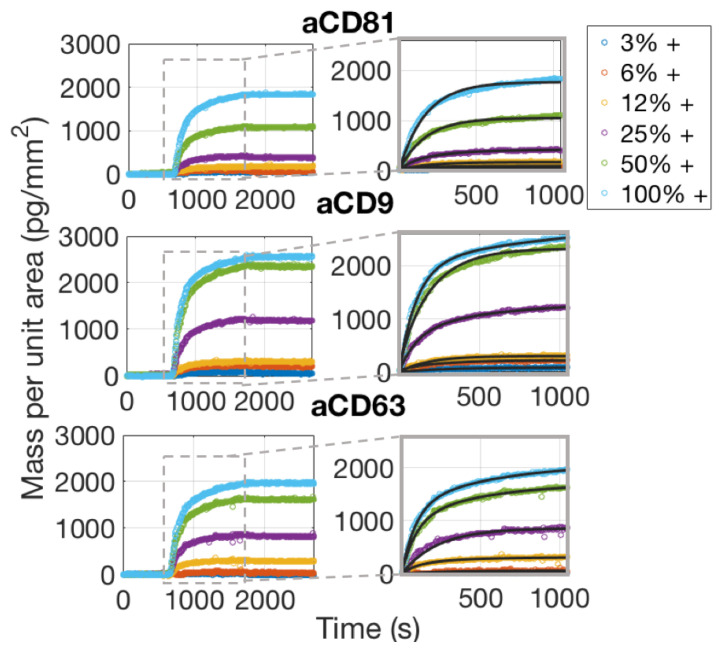
Binding curves of a generic IgG1 to one of the antibody chips used for the experiments. The insets focus on the association phase of the curves, which are fitted with a bivalent model.

The chip was first primed with buffer (PBS 1X) for 20 min, allowing for stabilization and acquisition of the baseline. Then, a generic IgG1 antibody was flowed across the surface for 20 min, at a flow speed of 200 uL/min. A subsequent wash with buffer was performed to allow for the dissociation phase. The binding curves obtained through this experiment are displayed in Figure 4. The curves were fitted with a bivalent model.

To derive the actual surface concentration of each antibody, we considered both the ratios of the initial slope and of the equilibrium points of the binding curves with those of the 100% spot. Those analysis are displayed in Figure S3. As expected, the data obtained from these two analyses are in good correlation, which implies that both could be used for an estimate of the active antibody concentration.

It can be noted that, while the actual concentration of aCD81 in the spots is pretty consistent with the spotted concentration (the correlation in Figure S3 is almost linear), the concentration of aCD63 and aCD9 seems to saturate with the spotted concentration. This is probably related to a lower immobilization yield of the antibodies or to the particular conformation of the spots. Nonetheless, we were able to retrieve the information of the actual concentration of the active antibody spots, necessary for the EVs capture experiment. Since the chips belong to the same spotting batch, they are indeed identical in terms of spotting conditions.

### 3.3. Reflectance Simulations

With the IRIS technique, the accumulation of biomass on the sensor surface changes the optical path length, which results in a change in the measured reflectance signal. The reflectance from the IRIS substrate was simulated to understand the change in reflectance signal that results due to the accumulation of EVs on the sensor surface.

**Figure 5:**
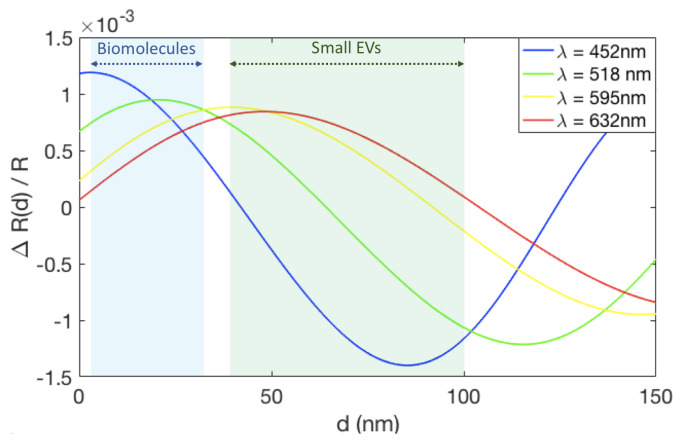
The change in reflectance signal due to biomass accumulation on an IRIS substrate with 110 nm of oxide for four different wavelengths, corresponding to the LEDs available on the IRIS system. The light blue region indicates the expected thickess range for biomolecules accumulation (1–30 nm), while the light green region shows the range expected for EVs (40–100 nm).

Figure 5 shows the change in reflectance signal in terms of the total reflectance (ΔR(d)/R) for a range of biomass accumulation (d) on an IRIS substrate with 110 nm of oxide. For this oxide thickness, the maximum change in reflectance signal is achieved using blue illumination (λ = 452 nm) for small amounts of biomass accumulation (0–20 nm). Since the small EVs are 50 to 150 nm in diameter, their accumulation on the surface surpasses the thickness where blue is optimal. Instead, green illumination (λ = 518 nm) shows a greater change in reflectance for this biomass accumulation range and was therefore chosen for these experiments.

### 3.4. Real-Time Specific Capture and Detection of EVs

The real-time characterization of an EVs sample was performed on the IRIS detection system by imaging different capture probe conditions simultaneously through the EVs solution in order to optimize incubation conditions. As mentioned above, the chips used for these experiments belong to the same spotting batch of those used for the control experiments, and are therefore assumed to have the same concentration of active probes on the surface. The chip was primed with 1X PBS for 20 min prior to injection of EVs. The EVs sample was injected and recirculated for 20 min at a flow speed of 200 μL/min. Images of the spots were acquired during the binding process and during the following buffer wash that was performed to operate the dissociation phase.

As is also confirmed by Single-Particle ExoView measurements (Section 3.1), aCD81 has a higher affinity for capturing EVs with respect to aCD63 and aCD9 and is therefore the most efficient capture probe.

**Figure 6:**
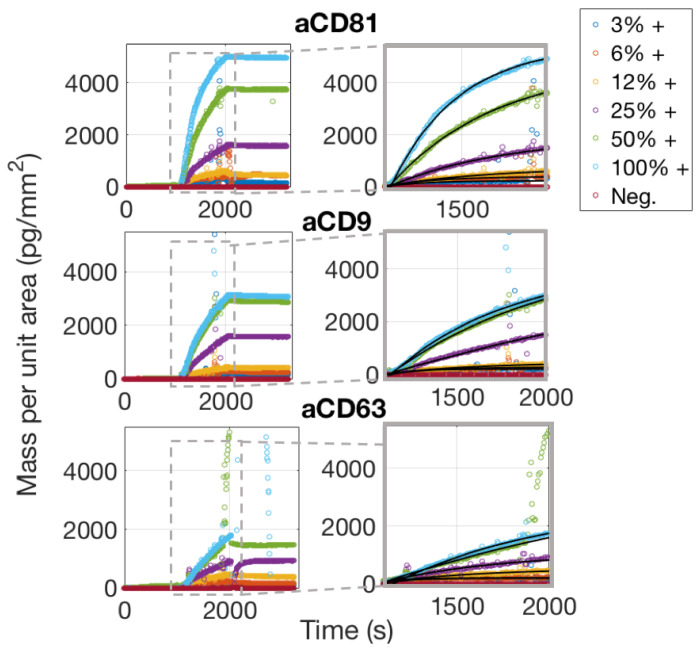
Simultaneously acquired real-time binding curves of EVs accumulating onto three different probes, each at six different concentrations. The insets focus on the association phase of the curves, which was fitted with a multivalent model that separates an initial, fast association rate $k_{on,fast}$ from a slower, subsequent rate $k_{on,slow}$ as explained in the Discussion Section 4.2.

Considering the dissociation phase of the curves in Figure 6, one would notice that the slope is almost flat, meaning that the vesicles are well anchored to the surface. This is expected, assuming that each vesicle possesses on its surface multiple receptors, which will therefore bind to multiple antibody sites. The appearance of the binding data and also the considerations stated above, clearly preclude the possibility of fitting the binding curves with a 1:1 Langmuir model. A multivalent model must be used, with an unknown number of binding sites to be considered per vesicle. We referred to the work by Li et al. [17] to derive a good model for the analysis of the binding curves of the exosomes to the surface probes. A more detailed discussion about the details of this fitting procedure are given in Section 4.2.

## 4. Discussion

### 4.1. Incubation Time Optimization: In-Flow Dynamic Capture

Time optimization of EVs experiments is tricky: most EVs characterization methods involve 12 h or overnight incubations with dyes and other probes, both for capture and, successively, staining. Maximizing the binding efficiency is a priority, and that requires time, especially if the incubations are carried out in a static condition.

An incubation process is defined as static when no flow is involved; instead, a small quantity of sample is deposited in the form of a droplet on the sensor, and the incubation is performed in a small humid chamber, which prevents the droplet from drying out. Obviously, this process only relies on diffusion mechanisms for the vesicles to explore the possible binding configurations. This method is widely used in DNA or protein fluorescence microarrays, and it works perfectly, since the binding agent is normally a molecule, and for most molecules, Brownian motion inside a droplet is enough to saturate all the binding sites in a very short time. However, EVs belong to the category of nanoparticles, and therefore, the Brownian motion they experience is significantly lower than what molecules would in the same conditions. This implies that it takes a longer time for the particles to diffuse to the surface, explore the possible binding configurations, and, eventually, bind.

Here, we demonstrate that a constant speed flow allows the particles to bind fast and efficiently: in a 20 min experiment, a high binding rate is achieved, especially on aCD81 spots. This result can be simply explained by considering how the flow helps the particles explore the available binding configurations in a more efficient way, maximizing their chance of finding a stable configuration on the surface. Moreover, even for high affinity binders, in our system, a flow rate of 200 μL/min is enough to minimize mass transport limitations [20]. Therefore, we demonstrate that a controlled flow system would improve the binding efficiency and therefore reduce the incubation time, for any EVs-related experiment.

### 4.2. Probe Density Optimization: Avidity Effects

Avidity effects occur when a nanoparticle that expresses more than one receptor molecule on its surface is captured on a surface that has been functionalized with a specific capture probe. Because of the multivalent nature of the reaction, the binding of the particle to the capture surface results in the occupation of more than one probe site, therefore limiting the possibility for other particles to bind in its vicinity. Avidity therefore describes a mechanism of multivalent binding of multiple ligands to multiple probes, and-depending on its range,-it can improve or diminish the binding efficiency of the nanoparticles.

**Figure 7:**
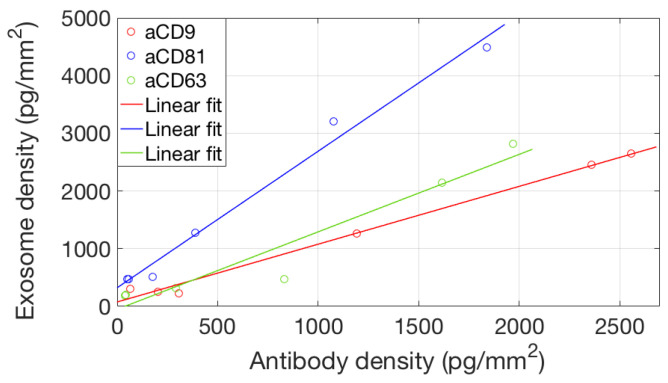
Correlation between the amount of active antibody immobilized on the surface and the total amount of captured EVs. The x coordinate of the data points corresponds to the maximum binding signal reached in Figure 4, while the y coordinate is the maximum EVs binding signal as shown in Figure 6. A linear correlation of the two datasets (no saturation) implies that—for this concentration range—the avidity regime does not change.

To study avidity effects in our system, we considered binding of EVs to different concentrations of the active probes. We considered the equilibrium binding signal from the EVs sample against the antibody surface density, and we found (Figure 7) a good linear correlation, suggesting that the avidity regime does not change in this probe concentration range.

However, the avidity of a nanoparticle also characterizes the binding behavior and the shape of the binding curve. Li et al. [17] characterize the binding characteristics of nanoparticles by considering a percentage of fast binders and slow binders. Fast binders are defined as the particles that first reach the surface, when all the binding sites are completely available, and can therefore quickly find a configuration that allows them to bind in a fast, stable way, by occupying as many binding sites as possible. We define the binding rate of fast binders as $k_{ON,fast}$. On the other hand, slow binders are defined as the particles that reach the sensor surface at a later time, when more than half of the available binding sites are already occupied and, therefore, have a harder time finding a configuration that allows them to stably bind. When they bind, they will do so at a lower rate ($k_{ON,slow}$), and each particle will occupy a lower number of binding sites with respect to a fast binder. Obviously, during the dissociation phase, fast binders—which are, by definition, bound in a more stable way—will be less prone to leave the surface with respect to slow binders, which instead occupy a smaller number of binding sites. Therefore, in this case, the slow binders will dissociate at a higher rate, while the fast binders will dissociate at a lower rate.

To apply this model to fit the binding curves, we simulated binding curves by considering different percentages of fast binders and slow binders in the sample, each of them having its own set of association and dissociation constants, for a total of five different parameters ($k_{ON,fast}$, $k_{OFF,fast}$, $k_{ON,slow}$, $k_{OFF,slow}$, $\rho_{fast2slow}$). The parameter $\rho_{fast2slow}$ is defined as the percentage of fast binders in the sample, and in our simulations, we varied it from 0 to 100% ($\rho_{fast2slow}$ = 0 (no fast binders) to $\rho_{fast2slow}$ = 1 (all fast binders)). The result of the simulations is displayed in Figure S4a. Here, we chose to vary the ratio of fast to slow binders because the amount of fast binders in the sample depends on the affinity of the probes, the size of the particles, the number of multivalent sites on each particle and the concentration of the sample. We then utilized the results of the simulations to decide which ratio of fast to slow binders was the most appropriate to fit our binding curves. The fitted binding curves for EVs binding to aCD9 and aCD81 spots are displayed on Figure S4b. Here, we chose a ratio of $\rho_{fast2slow}$ = 0.4, meaning 40% of fast binders. Clearly, the model works well for this case, and the fitted data have an $R^{2}=0.998$. The obtained binding constants are in the order of the ones utilized for the simulations, which are $k_{ON,fast}=10^{4}\;$M${}^{-1}$s${}^{-1}$, $k_{OFF,fast}=10^{-5}\;$s${}^{-1}$, $k_{ON,slow}=10^{3}\;$M${}^{-1}$s${}^{-1}$, $k_{OFF,slow}=10^{-10}\;$s${}^{-1}$.

## 5. Conclusions

We successfully demonstrated a label-free, multiplexed method for the optimization of the experimental parameters related to EVs experiments. This method would potentially allow researchers in the field of EVs characterization to dramatically reduce incubation time for EVs experiments, as well as optimize surface probe density, type of probe, and other experimental parameters. Simultaneous acquisition of binding of EVs to many different probes at different concentrations allowed for affinity characterization of the antibodies. A simplified multivalent model was used to analyze and fit binding curves of EVs, as detailed in Section 4.2. As expected, high affinity of EVs to aCD81 was demonstrated and also confirmed by Single Particle Interferometric Imaging measurements.

## Data Availability

Not applicable.

## Supplementary Materials

The following are available at https://www.mdpi.com/article/10.3390/s21082634/s1, Figure S1: A scheme of the IRIS setup, Figure S2: The fluorescence signal obtained for the analyzed EVs from staining the cargo with four different antibodies, Figure S3: Verification of the amount of the active antibody on the surface, Figure S4: Comparison of simulated and real binding curves of EVs.
