# Supplementary material for: Multiplexed Affinity Measurements of Extracellular Vesicles Binding Kinetics
Source: Sensors (Basel). 2021 Apr 9;21(8):2634. doi: 10.3390/s21082634 (PMC8069658; doi:10.3390/s21082634)
Supplement: Supplementary file 1 [file sensors-21-02634-s001.pdf]

Real-time measurements of extracellular vesicles binding kinetics in a multiplexed microarray modality

Supplementary Information

Authors:

Elisa Chiodi
Department of Electrical Engineering
Boston University, Boston (MA)

George Daaboul
Nanoview Biosciences
Boston (MA)

Allison M. Marn
Department of Electrical Engineering
Boston University, Boston (MA)

M. Selim Ünlü
Department of Electrical Engineering
Boston University, Boston (MA)

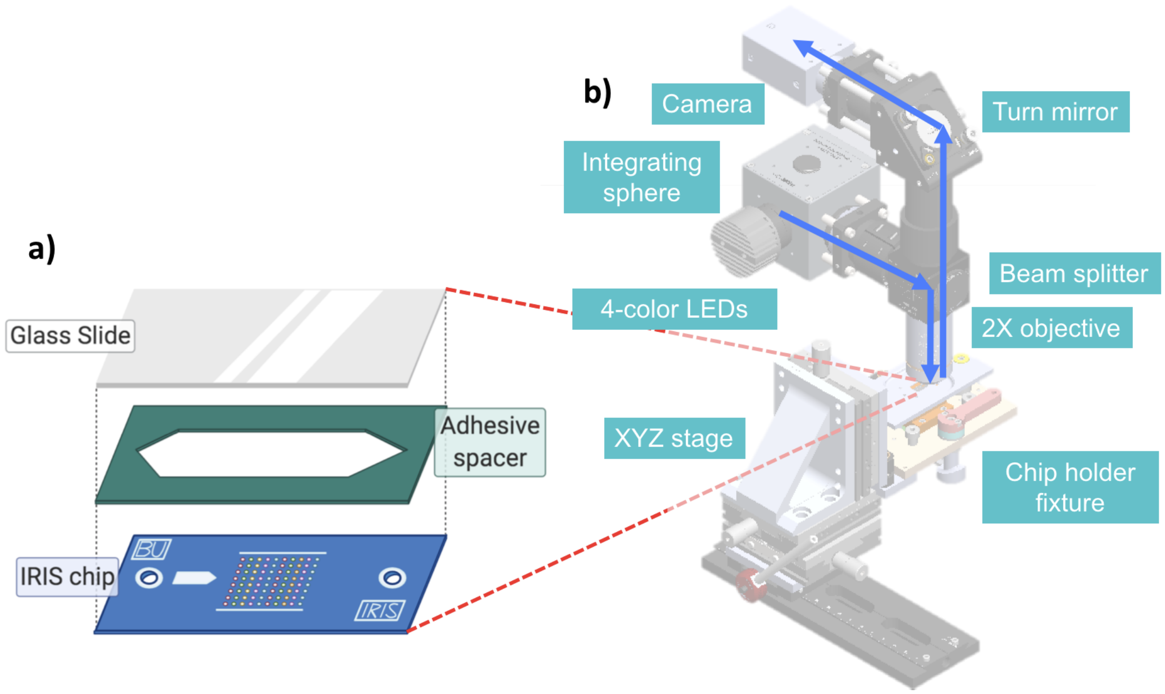

Figure S1: A scheme of the IRIS setup. a) The microfluidic chamber composed of the chip, with laser-drilled inlet and outlet holes, an adhesive spacer and a glass slide. b) A rendering of the common-path interferometer IRIS setup utilized for the experiments, where the light path is highlighted with blue arrows. The red dotted lines show the position of the microfluidic chamber, kept in place by a clamping fixture.

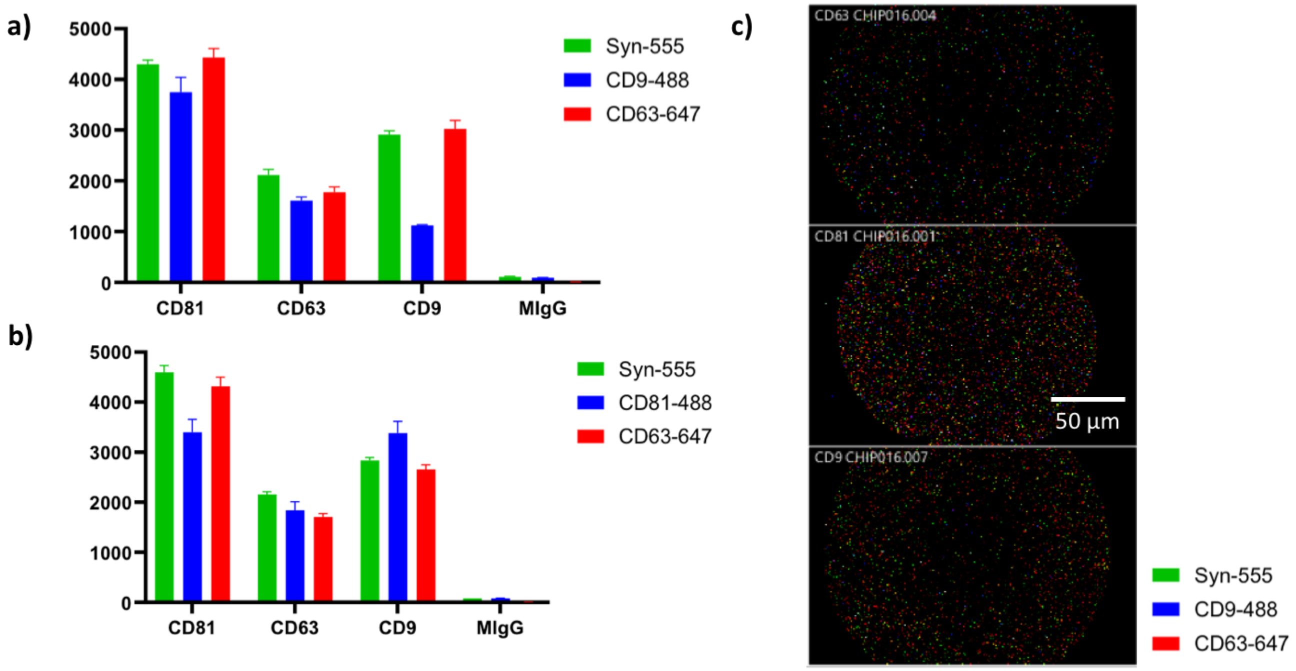

Figure S2: The fluorescence signal obtained for the analyzed EVs from staining the cargo with Syn-555, and the membrane with a) CD9-488, CD63-647 or b) CD9-488 and CD81-488, as they are captured on three different membrane-specific probe spots (aCD9, aCD63, aCD81). c) A co-localized fluorescence image of three different capture spots on one of the chips used for the test.

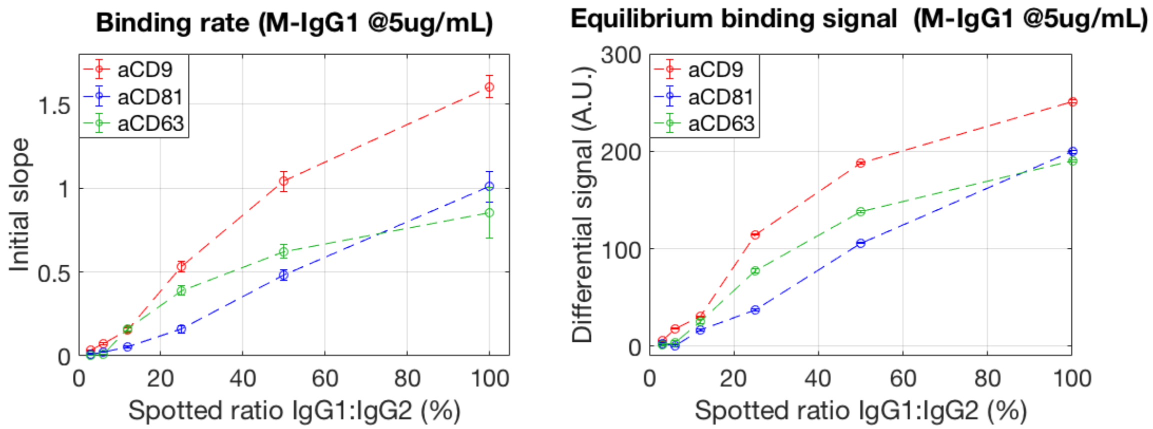

Figure S3: Verification of the amount of active antibody on the surface. The spotted percentage of active probe versus a) the initial slope of the binding curves in Figure 4 and b) the maximum signal obtained on the same dataset.

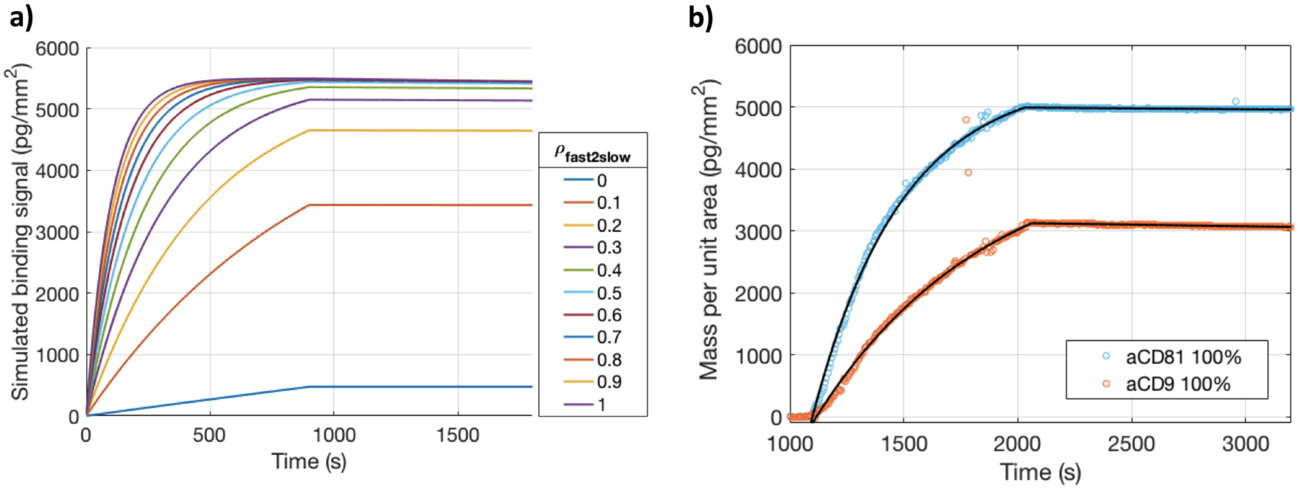

Figure S4: Comparison of simulated and real binding curves of EVs. a) Simulated curves, where increasing percentages of fast binders versus slow binders are considered. Here, $k_{ON,fast} = 10^4 M^{-1}s^{-1}$, $k_{OFF,fast} = 10^{-5}s^{-1}$, $k_{ON,slow} = 10^3 M^{-1}s^{-1}$, $k_{OFF,slow} = 10^{-10}s^{-1}$ at different percentage of fast and slow binders. model utilized for fitting EVs binding curves. The black line indicates the fit.
